# Supplementary material for: VGLUT1 functions as a glutamate/proton exchanger with chloride channel activity in hippocampal glutamatergic synapses
Source: Nat Commun. 2017 Dec 22;8:2279. doi: 10.1038/s41467-017-02367-6 (PMC5741633; doi:10.1038/s41467-017-02367-6)
Supplement: Supplementary file 1 — Supplementary Information [file 41467_2017_2367_MOESM1_ESM.pdf]

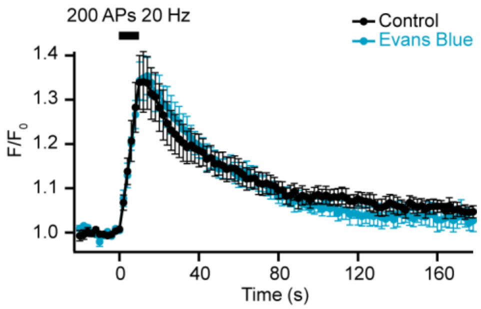

Supplementary Figure 1 The non-permeant inhibitor of VGLUT, Evans Blue, does not inhibit SV cycle or acidification Average SpH fluorescence response of glutamatergic boutons to 200 APs at 20 Hz with (blue) or without (black) 1 µM Evans Blue for 5 min (n = 5 experiments for each condition). Error bars represent s.e.m. from more than three neuronal preparations.

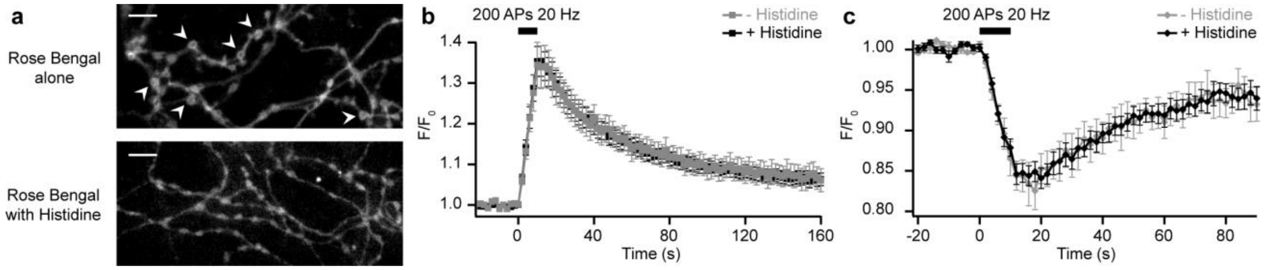

Supplementary Figure 2 Histidine does not impact exo-endocytosis of SVs (**a**) Fluorescence images of hippocampal axonal arborisations expressing SpH in presence of RB with or without histidine, during light illumination. Illumination of RB produces singlet oxygen inducing neurotoxicity in absence of the scavenger histidine. The swollen boutons (arrowheads) in the upper image do not respond to electrical stimulation (data not shown). The scale bars represent 5 µm. (**b**,**c**) Changes in SpH (**b**) and αVGAT-cypHer (**c**) fluorescence intensity in response to stimulation (200 APs at 20 Hz) in hippocampal neurons with (black, n = 24) or without (grey, n = 14) 10 mM histidine. Error bars represent s.e.m. from more than three neuronal preparations.

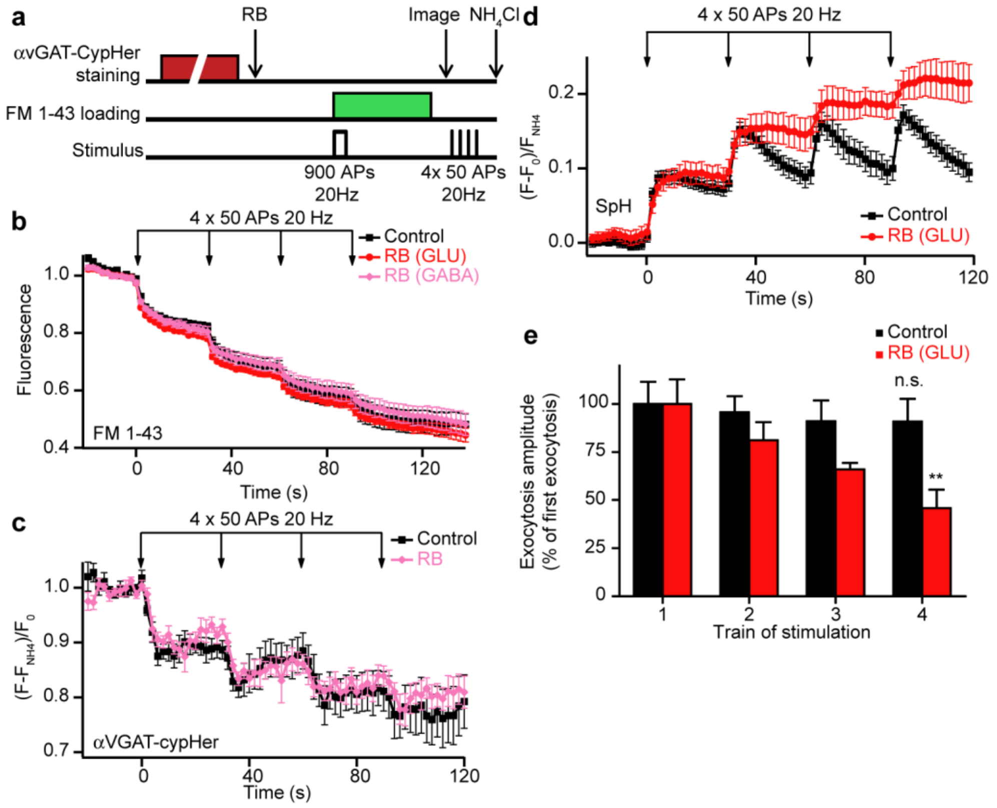

Supplementary Figure 3 VGLUT inhibition does not affect exo-endocytic cycling (**a**) Experimental protocol for labelling empty glutamatergic vesicles with FM1-43 and analysing their recycling. (**b**,**c**) Normal exo-endocytotic cycling of SVs in presence of RB. After loading, four bouts of 50 APs were delivered to the neurons causing FM1-43 destaining (**b**) and αVGAT-cypHer transients (**c**) (red: glutamatergic boutons, pink: GABAergic boutons, n = 4-6). (**d**,**e**) Reuse of inhibited glutamatergic SVs. Reduction of SpH response amplitude to consecutive trains of stimulations indicates exocytosis of non-quenched SpH, i.e. empty neutral vesicles (n = 12 for control conditions, 8 for glutamatergic boutons with RB). Average SpH and αVGAT-cypHer traces were normalized to the respective total pool size determined by a pulse of NH_4Cl at the end of the experiment. n.s., non significant, P = 0.5883 compared with first stimulation of control, analysed with two-tailed unpaired *t*-test; **, P = 0.0083 compared with first stimulation in presence of RB, analysed with two-tailed unpaired *t*-test. Error bars represent s.e.m. from more than three neuronal preparations.

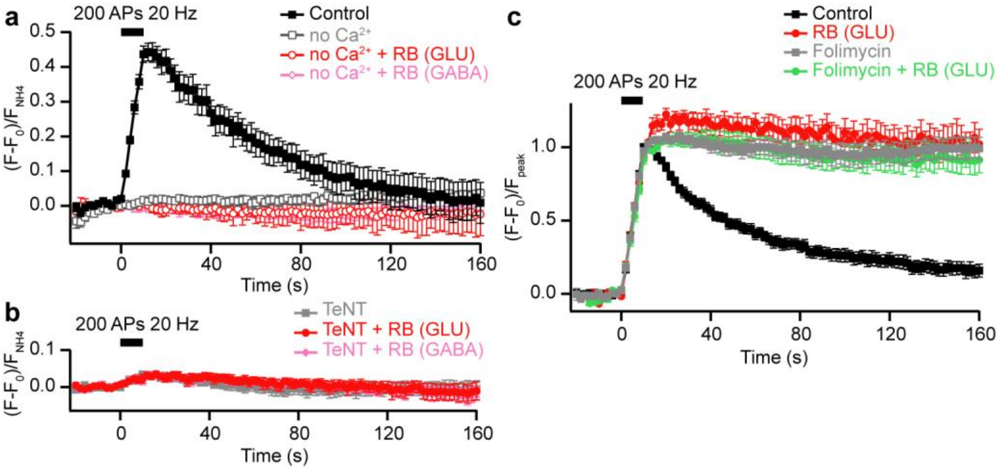

Supplementary Figure 4 RB-induced post-stimulus luminal alkalinisation requires exocytosis of V-ATPases (**a**) Average SpH fluorescence responses upon application of 200 APs at 20 Hz in control conditions (black), without Ca^{2+} (grey, 0 mM nominal Ca^{2+} + 2 mM EGTA) and without Ca^{2+} in presence of RB (red: glutamatergic boutons, pink: GABAergic boutons) (n = 4-9). (**b**) Average SpH fluorescence transients upon stimulation in presence of 10 nM Tetanus Neurotoxin for 16-18 h (TeNT, grey) alone or in combination with 100 nM RB for 5 min (red: glutamatergic boutons, pink: GABAergic boutons) (n = 8-11). In **a** and **b** average traces were normalized to the total pool size determined by a pulse of NH_4Cl at the end of the experiment. The post-stimulus SpH fluorescence increase induced by RB (Fig. 1f) was suppressed by chelation of extracellular calcium with ethylene glycol-bis(2-aminoethylether)-N,N,N',N'-tetraacetic acid (EGTA) (**a**) and by inhibition of vesicle fusion with tetanus neurotoxin (**b**). Therefore, the alkalinisation is dependent on exocytosis, but not related to the calcium increase during stimulation. (**c**) Post-stimulus increase in SpH fluorescence induced by RB is selectively blocked by inhibiting V-ATPase with 65 nM Folimycin (n = 7-13). Average traces were normalized to the fluorescence intensity at the end of the stimulation. Error bars represent s.e.m. from more than three neuronal preparations. These results indicate that the post-stimulus alkalinisation induced by RB is caused by exocytosis of V-ATPases. V-ATPase has been shown

to be inserted into the bouton membrane during synaptic exocytosis and to trigger a transient alkalinisation of the cytosol that outlasts the stimulation train[1]. The reported cytosolic alkalinisation kinetics match the kinetics of the observed SpH fluorescence increase. Taken together, a transient cytosolic alkalinisation appears to induce a slight transient SV alkalinisation in presence of RB, suggesting that VGLUT inhibition renders SV sensitive to cytosolic pH changes. Note that using the 'rapid acid quench' protocol (Fig. 6) no post-stimulus alkalinisation was observed in the presence of RB, presumably due to the acidic extracellular environment which inhibited V-ATPase activity at the plasma membrane.

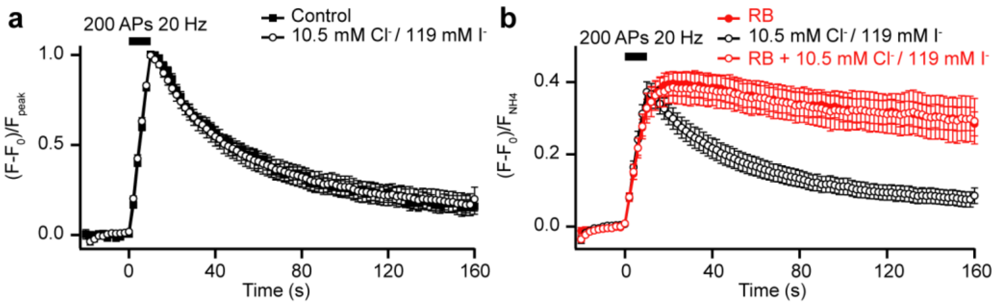

Supplementary Figure 5 SV cycling is not affected by substitution of chloride by iodide (**a**,**b**) Normalized SpH fluorescence response of glutamatergic boutons to 200 APs at 20 Hz in presence of normal Cl^- concentration (129.5 mM) or a combination of 10.5 mM Cl^- and 119 mM I^- with or without RB (100 nM, 5 min). Traces in A were normalized to the peak after stimulation. Traces in **b** were normalized to the total pool size uncovered by a pulse of NH_4Cl at the end of the experiment (n = 9-16). Error bars represent s.e.m. from more than three neuronal preparations.

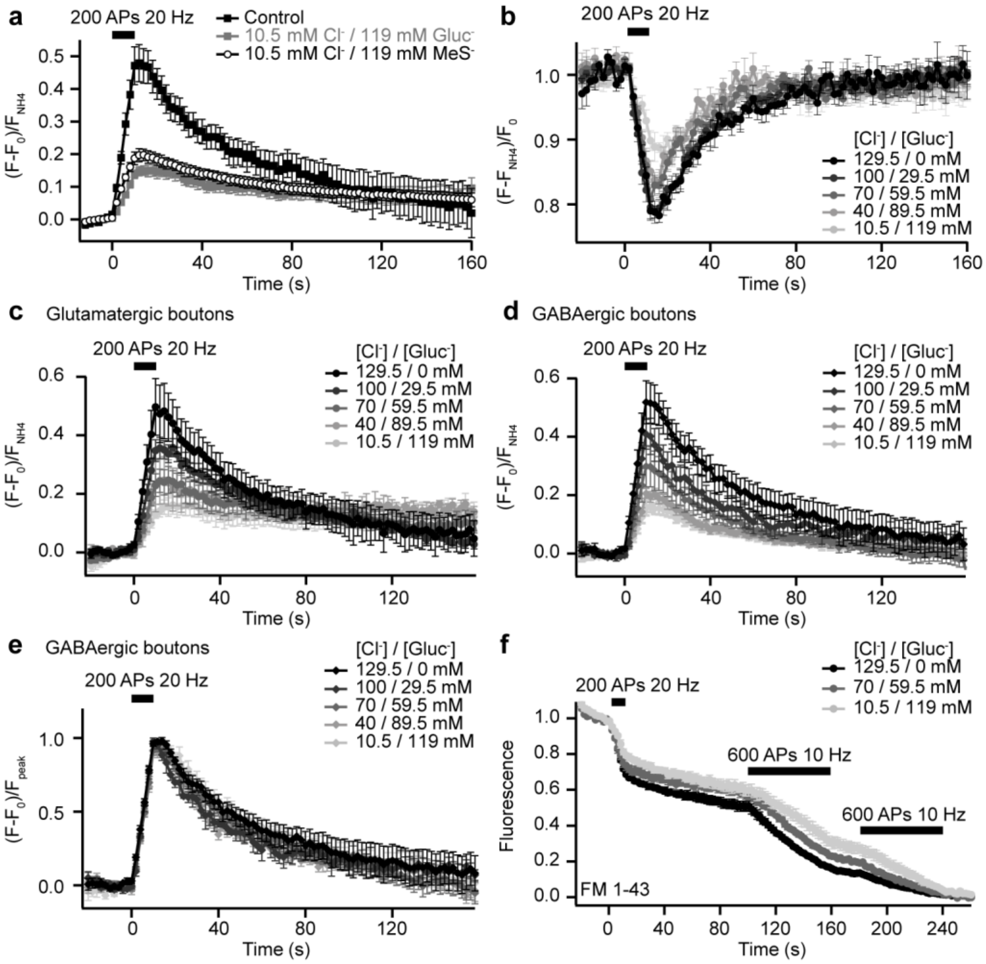

Supplementary Figure 6 Reduced extracellular chloride concentration impairs neuronal excitability (**a**) SpH fluorescence responses to stimulation (200 APs 20 Hz) in presence of 129.5 mM Cl^- and after substitution of 119 mM Cl^- by $Gluc^-$ or by MeS^- (n = 7-16). (**b**) αVGAT-cypHer is used to distinguish glutamatergic (**c**) from GABAergic (**d**,**e**) hippocampal neurons exposed to different combinations of extracellular [Cl^-]/[$Gluc^-$] (n = 3-10). Average traces in **a**-**d** were normalized to the total pool size determined by a pulse of NH_4Cl at the end of the experiment. Average traces in **e** were normalized to the peak after stimulation. (**f**) Loading of FM1-43 at normal Cl^- concentration followed by stimulation in presence of different combinations of [Cl^-]/[$Gluc^-$] reveals the effect of Cl^- concentration on exocytosis amplitude (n = 5-6). Note, that the effect of Cl^- substitution on exocytosis is systematically underestimated in FM1-43 experiments due to the zero offset adjustment necessary for normalization. Error bars represent s.e.m. from more than three neuronal preparations.

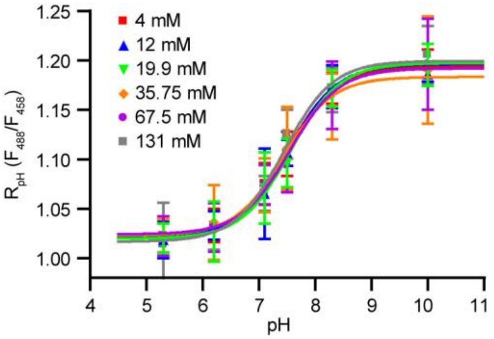

Supplementary Figure 7 Calibration of SytClopH Variation of Cl^- concentration within the physiological range (4-131 mM) had no effect on RpH. Error bars represent s.e.m. from more than three neuronal preparations.

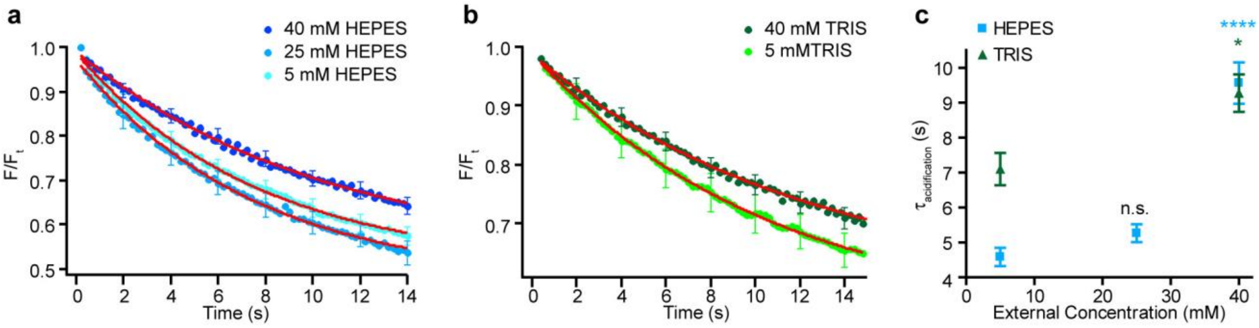

Supplementary Figure 8 Influence of external proton buffers on SV acidification kinetics (**a**,**b**) Rapid acid quench of surface SpH fluorescence following stimulation (200 APs at 20 Hz) in presence of different HEPES (**a**) or TRIS (**b**) concentration (n = 20-51). Traces represent only the post-stimulus acid quench period. They are normalized to the first data point measured during this quench and are fitted with single exponential. (**c**) Plot of reacidification time constants versus external HEPES or TRIS. n.s., non significant compared with 5 mM HEPES; ****, p <0.0001 compared with 5 and 25 mM HEPES; *, p = 0.0122 compared with 5 mM TRIS, analysed with two-tailed unpaired *t*-test. Error bars represent s.e.m. from more than three neuronal preparations.

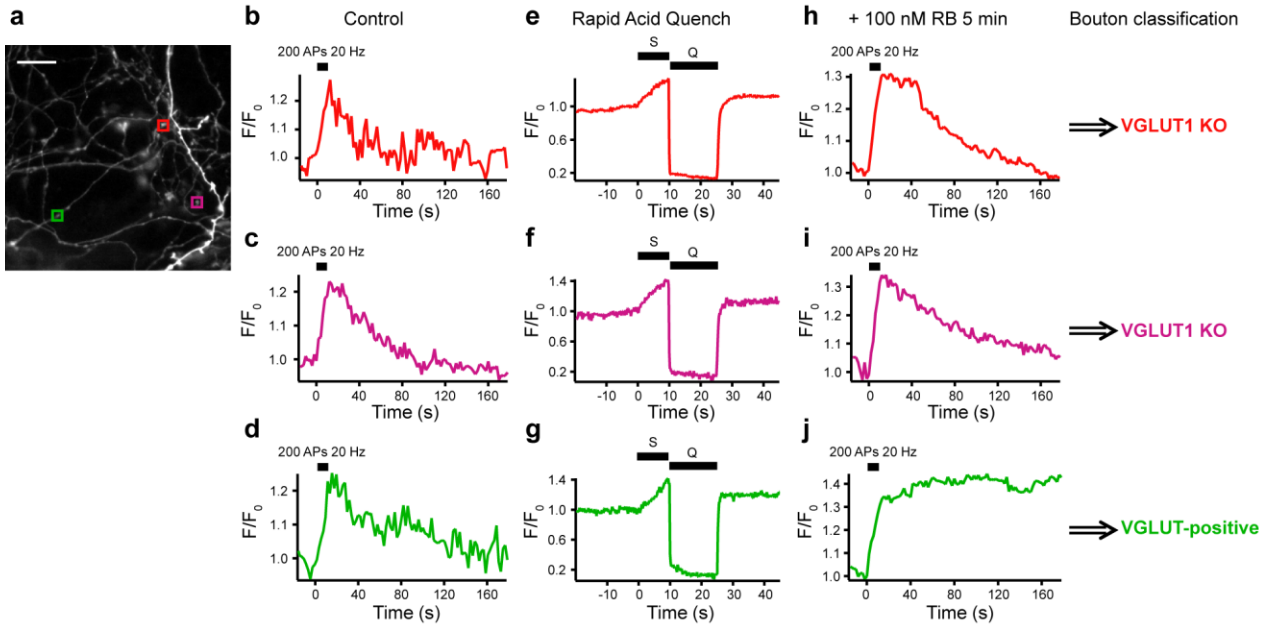

Supplementary Figure 9 Sorting of VGLUT1-deficient and VGLUT-positive boutons A subpopulation of wild-type hippocampal neurons co-expresses VGLUT1 and VGLUT2 (ref 6) prompting us to sort out VGLUT-positive boutons in VGLUT1-deficient mice. (**a**) Fluorescent image of SpH-transfected hippocampal neurons from VGLUT1-knockout (KO) mice. To explain the sorting process, three boutons which didn't load αVGAT-CypHer and are thus glutamatergic, are selected and analysed (**b**-**j**). Hippocampal neurons were first challenged with 200 APs at 20 Hz (**b**-**d**) showing exo-endocytosis cycling similar to wild-type neurons. Then, the 'rapid acid quench' strategy was applied to monitor directly the re-acidification kinetics (**e**-**g**). Finally, neurons are incubated for 5 min with 100 nM RB and stimulated with 200 APs at 20 Hz (**h**-**j**). The inhibited bouton (**j**) contain VGLUT and show a re-acidification kinetic (**g**) similar to wild-type ones (Fig. 6). The non-inhibited boutons (**h**,**i**) are devoid of any VGLUT and show a fast re-acidification (**e**,**f**).

Supplementary Table 1 Sequences of primers used to generate the synaptotagmin 1-ClopHensorN (SytClopH) construct

Name of the primer	Sequence (5'-3')
SDM_ClopH_HindIII_fwd	CCGCGCCACCAAGCTTAGCTGGAGCC
SDM_ClopH_HindIII_rev	GGCTCCAGCTAAGCTTGGTGGCGCGG
ClopH_HindIII_fwd	CCGCGCCACCAAG
ClopH_XmaI_rev	CCCGGCCCGGGACCGTACAGGAACAGG

Supplementary reference

1. Zhang, Z., Nguyen, K. T., Barrett, E. F. & David, G. Vesicular ATPase inserted into the plasma membrane of motor terminals by exocytosis alkalinizes cytosolic pH and facilitates endocytosis. *Neuron* **68,** 1097-1108 (2010).
